# Translational signatures and mRNA levels are highly correlated in human stably expressed genes

**DOI:** 10.1186/1471-2164-14-268

**Published:** 2013-04-19

**Authors:** Sergio R P Line, Xiaoming Liu, Ana Paula de Souza, Fuli Yu

**Affiliations:** 1 Piracicaba Dental School, University of Campinas, PO Box 52, Piracicaba, SP, 13414-903, Brazil; 2 The Human Genome Sequencing Center, Baylor College of Medicine, Houston, TX, 77030, USA; 3 Human Genetics Center, School of Public Health, The University of Texas Health Science Center at Houston, Houston, TX, 77030, USA

## Abstract

**Background:**

Gene expression is one of the most relevant biological processes of living cells. Due to the relative small population sizes, it is predicted that human gene sequences are not strongly influenced by selection towards expression efficiency. One of the major problems in estimating to what extent gene characteristics can be selected to maximize expression efficiency is the wide variation that exists in RNA and protein levels among physiological states and different tissues. Analyses of datasets of stably expressed genes (i.e. with consistent expression between physiological states and tissues) would provide more accurate and reliable measurements of associations between variations of a specific gene characteristic and expression, and how distinct gene features work to optimize gene expression.

**Results:**

Using a dataset of human genes with consistent expression between physiological states we selected gene sequence signatures related to translation that can predict about 42% of mRNA variation. The prediction can be increased to 51% when selecting genes that are stably expressed in more than 1 tissue. These genes are enriched for translation and ribosome biosynthesis processes and have higher translation efficiency scores, smaller coding sequences and 3^′^ UTR sizes and lower folding energies when compared to other datasets. Additionally, the amino acid frequencies weighted by expression showed higher correlations with isoacceptor tRNA gene copy number, and smaller absolute correlation values with biosynthetic costs.

**Conclusion:**

Our results indicate that human gene sequence characteristics related to transcription and translation processes can co-evolve in an integrated manner in order to optimize gene expression.

## Background

The control of gene expression is one of the most important biological processes, which can be regulated at diverse steps. Gene expression is the most energetically expensive process within a cell, and an efficient usage of gene expression machinery is of key importance for proper cell functioning. The rates of gene expression are mainly determined by the DNA sequences that modulate transcription and translation processes [1], and the selection for efficient ribosome usage seems to be a major force that shapes the evolution of gene sequences towards optimum gene expression [2]. Optimum gene expression can be understood as the maximum possible ratio between the benefit due to expression of the gene at a determined level and the costs of its production [3]. Optimum gene expression requires a balanced contribution of the diverse processes that control this process, as a gene that is transcribed at high rates should also be efficiently translated [4]. Therefore, it is expected that gene sequence characteristics that participate in the different aspects of gene expression machinery will work in an integrated manner and co-evolve in order to cope with demands for optimum ribosome usage.

Analysis of diverse prokaryotic and invertebrate species showed significant correlations between gene characteristics related to expression, such as codon usage [5-7], gene size [8,9], and folding energy at 5′ of mRNA [10]. Due to the relative small population sizes, the gene sequences of human, and other mammalian species, are not expected to be strongly influenced by selection towards expression efficiency [11,12]. In fact, except for gene and CDS sizes, there seems to exist a weak correlation between gene expression and specific gene signatures in humans, and higher correlation values are only obtained when analyzing pooled groups containing several hundred genes in each group [13-15].

One of the major problems in estimating to what extent a specific gene feature has undergone selection to maximize expression efficiency in humans is the wide variation that exists in RNA and protein levels among distinct tissues and even within the same tissue in different physiological and pathological conditions. In this sense, studies on this subject matter would benefit from the use of datasets of stably expressed genes (i.e. with consistent expression between physiological states and tissues), as those would provide more accurate and reliable measurements of associations between variations of a specific gene characteristic and expression, and how a distinct gene signature contribute to optimize gene expression. In this study we analyzed the association of gene characteristics related to translation efficiency or speed and mRNA expression using a dataset that was curated specifically for stably expressed genes [16].

## Methods

### Datasets and sequences

The genes used in this work were subdivided in two groups as follow:

Group 1: Formed by a dataset of stably expressed genes [16]. The genes were strictly selected using uniform data preprocessing and data quality control of 4,804 Affymetrix HU-133A arrays performed in clinical samples. Details of gene selection and analysis can be obtained in the Material and Methods section of the above referred paper. The complete list with Affimetrix GeneChip expression intensity and variation was download from supplementary Data S1 file of reference 16 (n = 575).

Group 2: formed by genes of Group 1, which were expressed in at least 2 tissues and had a/standard deviation/mean mRNA expression values < 0.4 (n = 196).

Group 3: formed by genes of Group 1, which were expressed in at least 3 tissues and had a standard deviation/mean ratio of mRNA expression values < 0.4 (n = 99).

Group 4: A list of genes with determined mRNA and Protein concentrations, obtained from Vogel et al [17]. The mRNA and protein were extracted from the human medulloblastoma Daoy cell line. mRNA expression values were generated using NimbleScan expression Robust Multi-array Analysis. Details of gene selection and analysis can be obtained in the Material and Methods and Supplementary Information of the referred paper. The complete list with Protein and mRNA expression intensity and variation was downloaded from Supplementary Data File. This and the previously described dataset were made non-redundant as genes included in Group 1 dataset were removed from this dataset (Group 4 = Vogel et al dataset - Group 1, n = 503).

cDNA, coding, 5^′^ UTR, 3^′^ UTR sequences and gene sizes were obtained from Biomart [18], http://www.biomart.org. Sequences with “N” representing unknown bases were removed from analysis. Coding sequences that do not start with “ATG” or do not finish with a stop codon or whose sequence sizes are not divisible by 3 were also removed. The databases of sequences were made non-redundant with respect to alternative splice variants, as only the longest sequence was used to represent each gene sequence. Since we used measures of codon bias, genes that do not code for proteins were removed from the analysis.

### Gene information and characteristics

Gene ontology was used to analyze the gene functions of the datasets used in the present work [19], http://www.geneontology.org. A set of 21 Biological Process Terms were selected from the Refine Selection menu of QuickGO [20], http://www.ebi.ac.uk/QuickGO. The genes were downloaded from Biomart [18], http://www.biomart.org.

tRNA Adaptation index (tAi) was measured according to dos Reis et al. [7]. The relative adaptiveness values for all human codons were obtained from Waldman et al. [15]. Codon Adaptation index (CAI), GC and GC3 contents were obtained using the CAI server [21], http://genomes.urv.cat/CAIcal/E-CAI. Isoaccepting tRNA gene copy numbers were obtained from Lavner and Kotlar [22].

Since previous reports have shown correlations between amino acid and gene expression [22], we also performed correlation analysis of the frequencies of amino acids and mRNA levels. The frequency of amino acids weighted by expression was calculated according to Lavner and Kotlar [22] and the amino acids size/complexity scores were obtained from Dufton [23].

RNA folding energies of 50 bp fragments of specific gene regions were obtained with the RNAFold program [24] using default settings. Only the minimum-free-energy (dG) structure was taken into account. The regions analyzed were:

a) The first 50 bases of cDNA.

b) The bases -52 to -2 of 5^′^ UTR.

c) The first 50 bases of coding sequence.

d) The last 50 bases of coding sequence.

e) The last 50 bases of cDNA.

f) 50 random bases from cDNA.

### Statistical analysis

All comparisons and statistical analysis were performed using R Statistical Package version 2.12.1 (http://www.r-project.org).

## Results

### Correlations between gene structural parameters and gene expression are higher in stably expressed genes

We selected a number of parameters representing the characteristics of the gene sequences that could be influential to the expression variation: Coding sequence size, untranslated regions size, mRNA stability, translation efficiency measured by tAi and CAI indexes and amino acid frequencies. Our rationale is that if gene sequences are selected to maximize expression efficiency we would expect a significant correlation between gene transcription, represented by mRNA levels, and parameters related to translational. The results of Spearman correlation analysis between gene characteristics and mRNA levels are shown on Table 1. There was a significant negative correlation between mRNA levels, CDS size and 3^′^ UTR size in Group 1 and Group 4. tRNA adaptation index (tAi) showed a positive correlation with mRNA levels in Group 1 and Group 4. There was a significant positive correlation between mRNA levels and folding energy of the 5^′^ UTR mRNA sequences from -2 to -52 in Group 1. There were no significant correlations between 5^′^ UTR sizes of and mRNA levels. Group 1 showed higher correlation values with CDS length, and folding energy in the end of 5^′^ UTR of mRNA than Group 4. The correlation values among gene characteristics and mRNA levels of Group 1, and to a lower extent of Group 4, are higher than the values reported in the literature. The correlation values presented with stably expressed dataset (Group 1) are the highest reported up to the present date, and they indicate that selection for gene sequence characteristics towards expression efficiency in human genes may be more relevant than previously believed. Significant Spearman correlations between mRNA levels and frequency of amino acids in Group 1 (p < 0.05) were found for amino acids Cys, Asp, Gly, His, Ile, Lys, Leu, Met, Pro, Gln, Ser, Val, Trp (Additional file 1: Table S1). Except for Gly and Trp, the amino acids showing significant correlations in Group 1 also exhibited significant correlation in Group 4. The signals of the correlations for Groups 1 and 4 (positive or negative) were coincident for all 20 amino acids. The higher correlations in Group 1 were found for amino acids Ser (cor = -0.4, p < 2.2e-16), Lys (cor = 0.35 p < 2.2e-16) and Gln (cor -0.25, p = 6.7e-10). These values were higher than previously published correlations in mice [25] and human [17] data. In order to further analyze the correlations between amino acids usage and gene expression, and to compare our data with previously published results of human genes [22], we correlated (spearman rank) the frequency of amino acids weighted by expression with the isoaccepting tRNA gene copy numbers and amino acids size/complexity score, which gives an estimate of the biosynthetic cost of amino acids [22,23]. Notably, Groups 1, 2 and 3 showed a higher positive correlation with isoaccepting tRNA gene copy number for each amino acid than Group 4 and from the groups reported by Lavner and Kotlar [22]. Groups 1, 2, and 3 also showed smaller negative correlations scores between the frequency of amino acids weighted by expression and amino acids size/complexity (Table 2). There is a significant negative linear correlation (r = -0.55, p = 0.01) between amino acids size/complexity and isoaccepting tRNA gene copy number [22], where amino acids with more tRNA genes have also smaller biosynthetic costs.

**Table 1:** Correlation analysis between gene parameters and mRNA expression

**Group**	**3**^′^**UTR size**	**5**^′^**UTR size**	**CDS size**	**tAi**	**CAI**	**dG 5**^′^**UTRs**
**1**	−0.29(1.2e-12)	−0.05(0.23)	−0.46(<2.2e-16)	0.32(3.3e-15)	0.07(0.08)	0.12(0.004)
**2**	−0.39(1.3e-08)	−0.01(0.89)	−0.51(1.2e-14)	0.39(4.6e-08)	0.15(0.03)	0.2(0.005)
**3**	−0.34(4.7e-4)	−0.18(0.07)	−0.53(1.7e-08)	0.18(0.07)	−0.01(0.89)	0.022(0.82)
**4**	−0.26(4.5e-09)	0.01(0.11)	−0.23(2.3e-07)	0.40(<2.2e-16)	0.21(3.1e-06)	−0.02(0.20)
**ref 13**			−0.20(1.0e-12)			
**ref 14**			−0.18(<1.0e-04)			
**ref 15**				0.23 (<0.0003)		

Spearman rank coefficients (p values) between gene characteristics and mRNA expression.

**Table 2:** Correlations between amino acids frequencies weighted by expression and isoaccepting tRNA gene copy number, and amino acids size/complexity score

**Frequency weighted by expression**	**Isoaccepting tRNA gene copy number spearman (p value)**	**Amino acids size/complexity score spearman (p value)**
Group 1	0.69(8.0e-04)	−0.75(1.2e-04)
Group 2	0.69(6.7e-04)	−0.73(2.4e-04)
Group 3	0.69(8.1e-04)	−0.73(2.4e-04)
Group 4	0.65(1.8e-03)	−0.80(1.6e-05)
High exp*	0.56(1.0e-02)	−0.79(2.8e-05)
All genes*	0.58(7.8e-03)	−0.80(2.0e-05)

Spearman rank coefficients (p-values). *From reference 22.

The parameters with significant correlation values (*p* < 0.05) were used as variables in multiple linear regressions and tested for their combinatorial effects in gene expression variation. CAI was not included in this analysis since tAi, which is also an index of codon bias, produced higher correlation coefficients and smaller *p* values. Using Akaike information criterion (AIC) we determined the best fitted regression models for Groups 1 (stable expression) and 4. This analysis was performed with the step command from library MASS, and the penalty per parameter used was log(# parameters).

**Figure 1:**
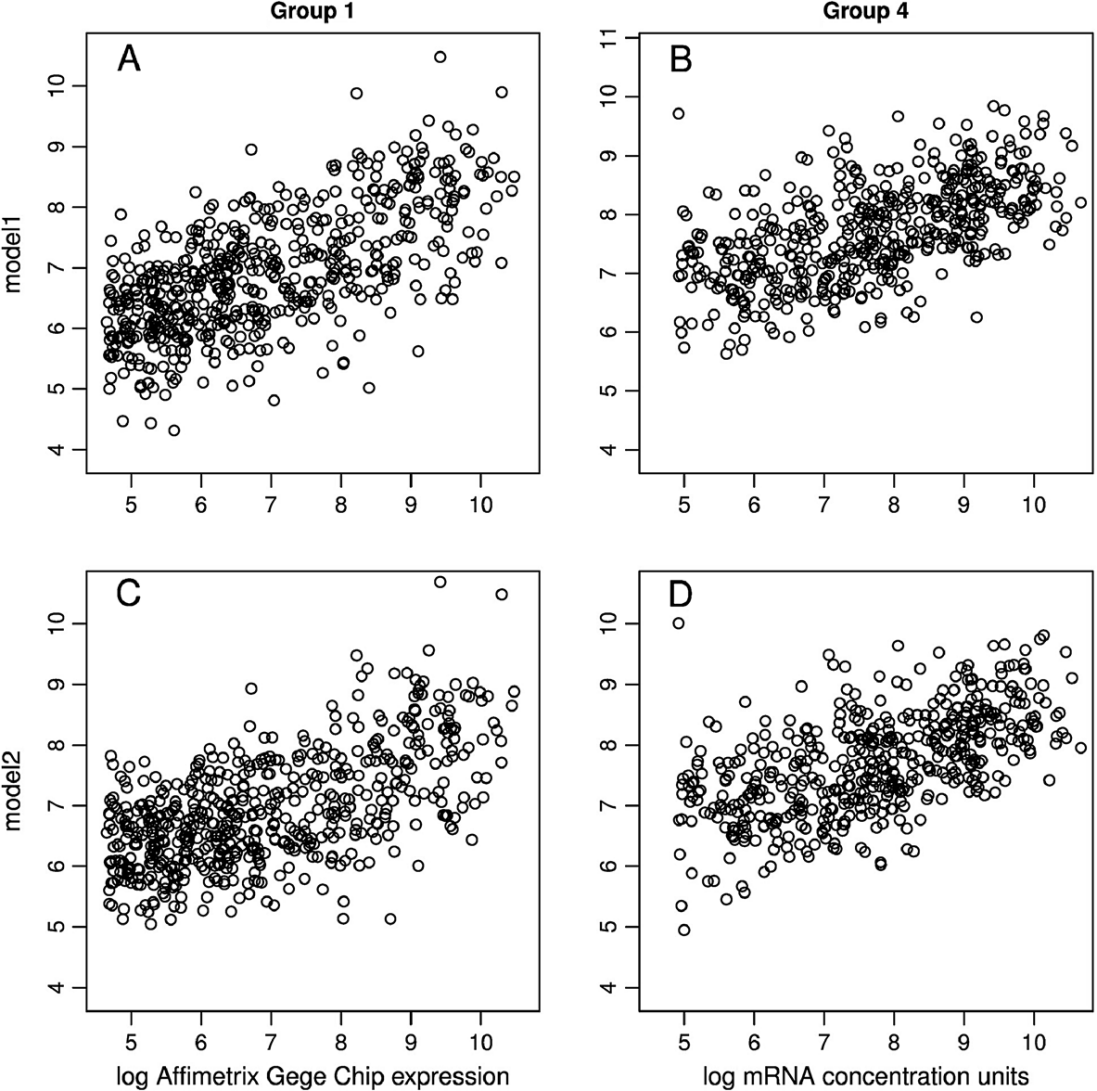
**Scatter plots of mRNA levels (x-axis) *vs *fitted model values (y-axis). A**. Regression of model1 in Group 1 (adjusted R-squared = 0.41, *p* < 2.2e-16, n = 575). **B**. Regression using variables from model1 in Group 4 (adjusted R-squared = 0.31, *p* < 2.2e-16, n = 503). **C**. Regression using variables from model2 in Group 1 (adjusted R-squared = 0.35, *p* < 2.2e-16, n = 575). **D**. Regression of model2 in Group 4 (adjusted R-squared = 0.33, *p* < 2.2e-16, n = 503).

The best regression model for Group 1 (model1, Table 3), included as independent variables the coding sequence length (Lcds), tRNA Adaptation index (tAi), length of 3^′^ UTR (L3utr), folding energy of the 50 bases from -2 to -52 of 5^′^ UTR (dG), and the frequencies of amino acids Cys, Glu, Leu, Gln, Ser, Asp. This model could predict 42% of mRNA variation in Group 1 (R-squared = 0.418, adjusted R-squared = 0.414, *p* < 2.2e-16, Figure 1A). Interestingly when tAi and log(Lcds) were replaced by the ratio tAi/log(Lcds) in model 1 there was a slight increase in the model performance (R-squared = 0.420, adjusted R-squared = 0.418, p < 2.2e-16).

**Table 3:** Summary of regression analysis (Group 1 & regression model1)

**Predictor**	**Coeficient (95% CI)**	**Significance**	Contribution*(95% CI)
Log(Lcds)	−0.36(-0.49,-0.23)	1.98e-07	0.26(0.19,0.33)
tAi	13.39(8.51,18.27)	1.09e-07	0.17(0.11,0.23)
Log(L3utr)	−0.25(-0.34,-0.16)	1.48e-07	0.16(0.10,0.22)
dG	0.05 (0.04,0.06)	7.40e-08	0.04(0.02,0.06)
Cys	−10.62(-18.41,-2.83)	0.00764	0.01(0.00,0.02)
Asp	−4.82(-10.04,0.40)	0.07043	0.01(0.00,0.02)
Glu	−5.55(-9.22,-1.88)	0.00316	0.02(0.01,0.03)
Leu	−10.37(-9.22,-1.88)	2.60e-08	0.06(0.03,0.09)
Gln	−16.13(-21.45,-10.79)	5.05e-09	0.08(0.04,0.12)
Ser	−10.37(-14.69,-6.05)	3.15e-06	0.19(0.14,0.24)

*Relative importance to log(expression) normalized to sum 100%. *Lcds* length of coding sequence.

*L3utr* length of 3^′^ UTR.

The best regression model for Group 4 included tAi, L3utr, dG and the frequencies of amino acids Cys, Glu, Leu, Gln, His, Arg, Tyr as independent variables (model2, Table 4). This model could predict 33% of mRNA variation in Group 4 (R-squared = 0.34, adjusted R-squared = 0.33, *p* < 2.2e-16, Figure 1D).

**Table 4:** Summary of regression analysis (Group 4 & regression model2)

**Predictor**	**Coeficient (95% CI)**	**Significance**	Contribution* (95% CI)
tAi	25.0(20.14,29.94)	< 2e-16	0.28(0.13,0.43)
Log(L3utr)	−0.14(-0.23,-0.05)	0.00355	0.17(0.08,0.26)
dG	0.02(0.01,0.03)	0.03023	0.01(0.00,0.02)
Cys	−22.39(-26.59,-18.20)	< 2e-16	0.18(0.10,0.26)
Glu	−5.34(-8.96,-1.72)	0.00396	0.01(0.00-0.02)
Gln	−10.58(-17.53,-3.63)	0.00292	0.06(0.01,0.11)
His	−14.64(-23.84,-5.44)	0.00189	0.21(0.10,0.32)
Leu	−7.92(-11.85,-3.99)	8.75e-05	0.05(0.01,0.09)
Arg	5.60(1.10,10.10)	0.01488	0.01(0.00-0.01)

*Relative importance to log(expression) normalized to sum 100%.

*L3utr* length of 3^′^ UTR, *dG* minimum folding energy. *tAi* tRNA adaptation index.

When variables from model1 were used in regression analysis in Group 4 dataset it was possible to predict about 35% of mRNA variation (R-squared = 0.36, adjusted R-squared = 0.35, p < 2.2e-16, Figure 1C), while variables of model2 in Group 1 could predict about 31% of mRNA variations (R-squared = 0.32, adjusted R-squared = 0.31, *p* < 2.2e-16, n = 503, Figure 1C). These results show that both regression models performed satisfactorily in both datasets and model1 produced higher adjusted R-squared values.

**Figure 2:**
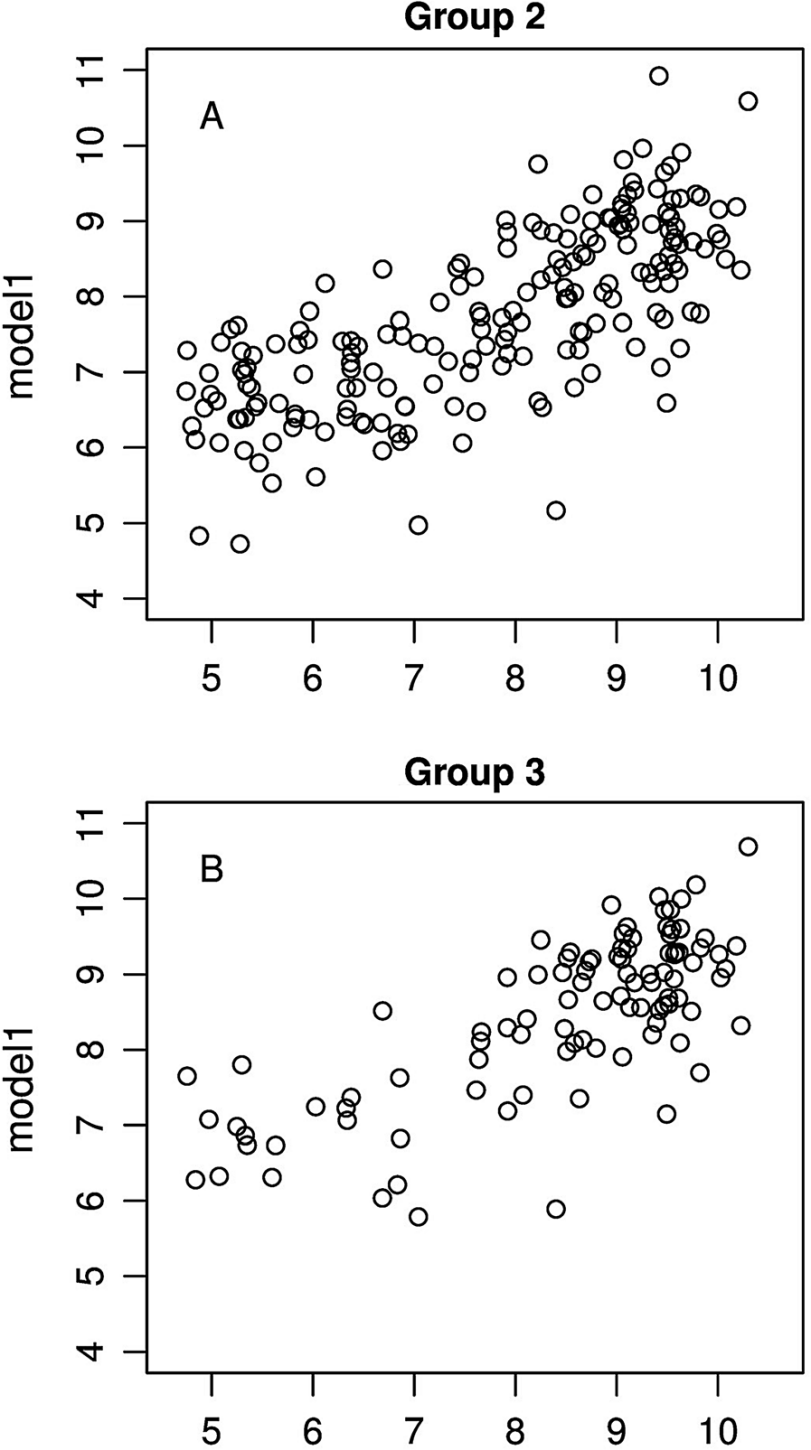
**Scatter plots of mRNA levels (x-axis) *vs *fitted model values (y-axis). A**. Regression using variables from model1 in Group 2, formed by genes that were expressed in at least 2 tissues and had a standard deviation/mean ratio of mRNA expression values < 0.4 (adjusted R-squared = 0.51, *p* < 2.2e-16, n = 196). **B**. Regression using variables from model1 in Group 3, formed by genes that were expressed in at least 3 tissues and had a standard deviation/mean ratio of mRNA expression values < 0.4 (adjusted R-squared = 0.50, *p* < 2.2e-16, n = 99).

The independent variables selected in model1 were used for regression analysis in two subsets of Group 1, comprised by genes stably expressed in at least 2 tissues and a standard deviation/mean ratio of mRNA expression values < 0.4 (Group 2, n = 196, Figure 2A), and with genes stably expressed in at least 3 tissues with a standard deviation/mean ratio of mRNA expression values < 0.4 (Group 3, n = 99, Figure 2B). It was possible to predict about 51% of mRNA levels in Group 2 (R-squared = 0.532, adjusted R-squared = 0.51, *p* < 2.2e-16), and about 50% of mRNA levels (R-squared = 0.553, adjusted R-squared = 0.50, *p* < 2.2e-16) in Group 3. tAi did not significantly contribute to the prediction of mRNA levels in Group 3, possibly due to little variation of tAi values within this group, which was formed by genes with the highest mRNA levels (see below and Table 5).

**Table 5:** Gene characteristics in the groups analyzed

**Grp**	3’ UTR size*	CDS size*	**tAi **^**+**^	dG*	mRNA*	**GO: Tr&RibSy**
**1**	981(842,1107)a	987(906,1028)a	0.367(0.36-0.37)a	−11.6(-12.4,-11.3)a	743(627,891)a	27.8% a
**2**	801(663,935)b	790(654,943)b	0.371(0.37-0.30)b	−10.9(-12.1,-10.9)b	3026(1891,4935)b	50.6% b
**3**	717(513,856)b	612(498,726)b	0.377(0.37-0.38)c	- 10.1(-11.3,-8.6)b	8212(5123,9010)c	69.0% c
**4**	1071(934,1226)a	1491(1356,1575)c	0.367(0.365-0.369)a	−12.0(-12.1,-10.8)a		18.0% d

* median (95% Confidence interval)l,^+^ mean:95% (Confidence interval), *Grp* Group, *dG* refers to minimum-free-energy of mRNA structure formed by 50 bases of 5′ UTR, *GO* Tr&RibSy refers to the % of genes belonging to the pool of genes that belong to translation or ribosome synthesis categories. Different letters (a, b, c, d) represent statistically significant differences (p < 0.05) among values in the same column. Differences in GO: Tr&RibSy were determined using chi-squared test for proportions with Yates’ correction.

These results clearly show a high association between mRNA levels and gene sequence characteristics related to translation in stably expressed datasets. These correlations indicate that sequence characteristics that modulate transcription and translation processes co-evolve in order to optimize ribosomal usage. This phenomenon, which has been underestimated due to the low correlations between gene expression and gene sequence characteristics reported in other studies, may have played a relevant role on the evolution of the human gene sequences.

### Stably expressed genes tend to have distinct structural characteristics and biological functions

Gene characteristics related to translation were compared using Kruskal. Wallis-test followed by post-hoc analysis using Mann–Whitney tests with Bonferroni correction. The results show that stably expressed genes (Groups 1, 2 and 3) tend to have smaller CDS lengths than “non-stably expressed” Group 4 dataset (*p* < 0.001). Group 1 and Group 4 had similar tAi values, while comparison among stably expressed Groups 1, 2 and 3 shows that the more tissues the gene is stably expressed the higher the tAi (*p* < 0.05). 3^′^ UTR sizes in Groups 2 and 3 were significantly smaller than in Groups 1 and 4. There was a small progressive decrease in folding energy when comparing Groups 4, 1,2, 3. Significant differences (p < 0.01), however, were only found between Group 4 and the other 3 groups. The highest differences were found when comparing the expression levels among Groups 1, 2 and 3, that showed a progressive ~4 fold increase in mRNA levels (*p* < 0.001) (Table 5).

The analysis of gene ontology showed that most genes in our datasets belong to at least one of the 21 Biological Process Terms used (Additional file 1: Table S2). Genes related to cell transport were the most frequent in Groups 4 and 1, while genes related to translation were the most frequent in Groups 2 and 3. The highest variations were seen in genes related to translation process and ribosome biosynthesis, whose frequencies increased progressively in groups 4, 1, 2, 3. Since translation and ribosome biogenesis categories are related, their genes were pooled and compared with the pool of genes present in the remaining 19 categories. Since a gene can be present in more than one category the genes present in the two pools were rendered unique (i.e. repeated genes were counted only once), and the genes present in the translation and ribosome biogenesis categories were removed from the second pool formed by the genes present in the remaining 19 categories. After this procedure the frequencies of translation and ribosome biosynthesis genes pool in groups 4, 1, 2 and 3 were 18.02% (62/344), 27.82% (111/399), 50.62% (81/170), 68.97% (60/87) (Table 5). The frequencies between each pair of groups are significantly different (Pearson chi-squared test with Yates’ correction, largest *p* < 0.009). These results show that stably expressed genes tend to be smaller than genes in the other two databases and are enriched for genes directly related to protein synthesis. These two characteristics are likely to be connected to gene expression levels, since genes related to translational machinery are frequently highly expressed, and highly expressed genes tend to have small size.

## Discussion

In the present paper we show that by using a dataset of stably expressed genes it is possible to predict about 42% of mRNA variation using 8 independent variables composed by gene characteristics related only to translation process. The predicted variation can be increased to about 51% when selecting genes that are stably expressed in at least 3 tissues (Group ST3). Although our results are based on the analysis of a dataset of 1078 genes, which comprises about 3-4% of the human coding sequences, our results clearly demonstrate that gene sequence characteristics may contribute significantly to the optimization of gene expression in human cells. It is worth mentioning that the *p* values obtained for CDS size and tAi in the correlation analysis were comparable to other studies that used much larger datasets [13-15,26]. The comparative analysis of mRNA of groups 1, 2 and 3 showed that the higher the mRNA expression the higher the frequency of genes related to translation and ribosome biosynthesis. These gene classes comprise the majority of RNA within the cell. Gene ontology analysis of Group 4 showed that genes in this group were somewhat enriched for genes related to translation and ribosome synthesis (18.02%). This may explain the fact that the correlation values for this group were in general larger than those reported in other published datasets [13-15]. Therefore, although, our sample represents only a fraction of genes within the cell, it may represent a significant fraction of expressed mRNA. Interestingly, groups 2 and 3, which are highly enriched for highly expressed translation and ribosome biosynthesis genes have the highest correlations between amino acids frequencies weighted by expression and amino acids isoaccepting tRNA gene copy number, and the smallest absolute correlation values with biosynthetic costs. One possible explanation for these results is that high demands for translation efficiency may occur at the expense of other aspects such as higher biosynthetic costs.

Two measures of codon bias were used in our analysis, tAi and CAI. In both datasets tAi produced higher correlation scores and lower p values than CAI. While CAI and some other indexes that estimate the role of codon bias in translational efficiency are based on the frequency of synonymous optimal codons found in highly expressed genes [27], tAi considers the tRNA pool within the cell, in which each codon is assigned a value that corresponds to the disponibility of the corresponding tRNAs [7]. tAi is, therefore, a more direct measure of biological function than CAI and other indexes based on codon frequency. In mammals, the few reports showing significant effect of synonymous mutations in gene expression and/or function, have mainly attributed its effects to changes in mRNA conformation [28] and disruption of mRNA splicing [12,29]. The significant correlation between tAi and mRNA levels observed here suggests that synonymous mutations may have a more active and broad role on gene expression than previously believed. Although synonymous mutations have been classically considered as “silent”, since they not alter the amino acid sequences of proteins, recent evidences indicate that human synonymous SNPs are targeted by positive and negative selection and can modulate the phenotype in humans [30-32]. In fact, besides modulating the levels of protein products changes in translation efficiency can further affect protein function by altering protein folding [33].

The stability at the 5^′^ end of mRNA is related to the translation efficiency in unicellular organisms [10,33]. Among all 50 bp fragments of diverse regions tested for mRNA stability, the sequences from -52 to -2 of 5^′^ UTR gave the smallest *p* values on both, correlation and multiple regression analysis. Using *E. coli* and *S. cerevisiae,* The 3^′^ UTR of genes is usually larger than 5^′^ UTR, and usually contains elements involved in post-transcriptional regulation of gene expression. In most cases, the binding of proteins and miRNA to 3′ UTR seems to increase degradation of mRNA molecules. In fact, miRNA regulation of gene expression seems to be avoided by decreasing the length of 3^′^ UTR [34]. Interestingly, there is a significant correlation between the size of 3^′^ UTR and frequency of tandem repeat sequences (r = 0.73, p = 0.0001) [35]. This may be a strategy to adapt the size 3^′^ UTR in order to cope with changes in selective pressure for variations in gene expression, as tandem repeats are prone to size variation due to slipped-strand mispairing. Our results support the biological importance of 5^′^ and 3^′^ UTR regions for efficient gene expression in humans, as gene characteristics of these regions (*dG* + *log(3UTR)*) can explain about 14% of mRNA variation in Group 1.

The correlation between mRNA levels and CDS length could be explained by the correlation of the former with gene length, which is a characteristic related to translation (Spearman rank of log CDS length *vs* log gene length = 0.48, *p* < 2.2e-16). The regression models with these two variables, however, showed that gene size and CDS size independently contribute to the prediction of mRNA levels (not shown), suggesting that these gene characteristics can evolve with certain independence to cope with optimal gene expression.

There was a progressive increase in mRNA levels of about 4 fold when comparing the values of groups 1, 2 and 3, whereas the changes in the individual parameters used as independent variables in regression analysis was of lower intensity (i.e. 1.3 fold for CDS length, and 1.03 fold for tAi). This is likely to be related with the fact that the ratio of the two major correlating factors (tAi/log(CDS length) used as a single independent variable can explain about 26.3% of mRNA variation, indicating that the variations in these two characteristics occur in a concerted manner. In this scenario small variations in the diverse characteristics can lead to substantial variation in gene expression. Large variability increases the repertoire of individual differences, which may be positively selected in order to cope with demands for efficient ribosome usage without affecting the function of the final protein product that is mainly determined by secondary and tertiary structures of protein polypeptide.

The high prediction power of translation parameters of model1 in Groups 1, 2, and 3, evidence the concomitant evolution of gene signatures related to translational efficiency and transcription activity measured by mRNA levels. These results suggest that the evolution of coding sequences can be influenced by changes in non-coding sequences and vice-versa. Recent evidences indicate that selection for efficient ribosome usage is the central force in shaping codon usage at the genomic scale [2]. This relationship has been discussed by Gingold and Pilpel [3], where these authors argue that genes with higher mRNA levels would be using up more ribosomes, and thus are under stronger selection for global translation efficiency, therefore, presumably creating the correlation between mRNA levels and gene characteristics related to translation efficiency such as tAi, CDS, 3^′^ UTR sizes and stability of secondary structures formed by folding of mRNA sequences near translation initiation site. Selection for ribosomal usage efficiency would be stronger in highly expressed genes, due to higher energetic costs. It is well known that there is a strong negative correlation between the expression level of a protein and its rate of evolution [36,37]. This relationship is currently explained by protein misfolding [37,38] and misinteraction avoidances [39]. Our analyses indicate that ribosomal usage efficiency may also be a relevant factor that determines gene the evolution of coding sequences in human genes and also possibly in other vertebrates.

## Conclusion

Our results indicate that human gene sequence characteristics related to transcription and translation processes can co-evolve in an integrated manner in order to optimize gene expression.

## Competing interests

The authors declare that they have no competing interests.

## Authors’ contributions

FY and SRPL conceived and designed the research project. SRPL performed the computational and statistical analysis. APS participated in the design and helped to draft the manuscript. XL participated in the design and helped to draft the manuscript. FY coordinated the research project. All authors read and approved the final manuscript.

## Supplementary Material

Additional file 1: Table S1 Correlation analysis between aminoacids frequency and mRNA expression. **S2.** Gene ontology analysis in Groups 1, 2, 3 and 4. Click here for file
