# Supplementary material for: Translational signatures and mRNA levels are highly correlated in human stably expressed genes
Source: BMC Genomics. 2013 Apr 19;14:268. doi: 10.1186/1471-2164-14-268 (PMC3639913; doi:10.1186/1471-2164-14-268)
Supplement: Additional file 1: Table S1 — Correlation analysis between aminoacids frequency and mRNA expression. S2. Gene ontology analysis in Groups 1, 2, 3 and 4. [file 1471-2164-14-268-S1.doc]

| **Table S1. Correlation analysis between aminoacids frequency and mRNA expression.**   |  | Group 1 | Group 4 | | --- | --- | --- | | Ala | 0.08 (0.05) | 0.22 (3.5e-07) | | Cys | -0.15 (0.0002) | -0.26 (2.3e-09) | | Asp | -0.06 (0.17) | 0.15 (0.0005) | | Glu | -0.17 ( 0.0004) | -0.04 (0.38) | | Phe | 0.02 ( 0.5) | -0.02 (0.57) | | Gly | 0.14 (0.0005) | 0.05 (0.2) | | His | -0.12 ( 0.004) | -0.26 (1.4e-09) | | Ile | 0.18 ( 8.5e-06) | 0.15 (0.0008) | | Lys | 0.35 ( 2.2e-16) | 0.17 (0.00016) | | Leu | -0.22 ( 4.6e-8) | -0.1 (0.02) | | Met | 0.13 (0.001) | 0.2 (2.8e-06) | | Asn | 0.02 ( 0.5) | 0.028 ( 0.5) | | Pro | -0.19 ( 4.8e-6) | -0.18 (3.7e-05) | | Gln | -0.25 ( 6.7e-10) | -0.14 (0.002) | | Arg | 0.06 ( 0.13) | -0.007 ( 0.8) | | Ser | -0.4 ( < 2.2e-16) | -0.29 (1.6e-11) | | Thr | -0.06 (0.13) | -0.004 ( 0.9) | | Val | 0.12 ( 0.003) | 0.16 (0.0003) | | Trp | -0.07 ( 0.07) | -0.04 ( 0.3) | | Tyr | 0.03 ( 0.4) | 0.07 ( 0.1) | |  |  |  |  |  |  |  |  |  | -0.22( 4.6E-8 | 0.13(0.001 | 0.02( 0.5 | -0.19( 4.8E-6 | -0.25( 6.7E-10 | 0.06( 0.13 | -0.4( < 2.2e-16 | -0.06(0.13 | 0.12( 0.003 | -0.07( 0.07 | 0.03( 0.4 |
| --- | --- | --- | --- | --- | --- | --- | --- | --- | --- | --- | --- | --- | --- | --- | --- | --- | --- | --- | --- | --- | --- | --- | --- | --- | --- | --- | --- | --- | --- | --- | --- | --- | --- | --- | --- | --- | --- | --- | --- | --- | --- | --- | --- | --- | --- | --- | --- | --- | --- | --- | --- | --- | --- | --- | --- | --- | --- | --- | --- | --- | --- | --- | --- | --- | --- | --- | --- | --- | --- | --- | --- | --- | --- | --- | --- | --- | --- | --- | --- | --- | --- | --- | --- |
|  |  |  |  |  |  |  |  |  |  | -0.1(0.02 | 0.2(2.8e-06 | 0.028( 0.5 | -0.18(3.7e-05 | -0.14(0.002 | -0.007( 0.8 | -0.29(1.6e-11 | -0.004( 0.9 | 0.16(0.0003 | -0.04( 0.3 | 0.07( 0.1 |

Spearman correlation (p values)

Table S2. Gene ontology analysis in Groups 1, 2, 3 and 4

| Ontology | Group4 # | Group 4 freq | Group 1 # | Group 1 freq | Group 2 # | Group2 freq | Group 3 # | Group 3 freq |
| --- | --- | --- | --- | --- | --- | --- | --- | --- |
| DNA metabolic process | 11 | 0.02 | 42 | 0.05 | 20 | 0.06 | 8 | 0.04 |
| RNA processing | 54 | 0.08 | 77 | 0.1 | 30 | 0.09 | 16 | 0.09 |
| Anatomical structure formation | 21 | 0.03 | 16 | 0.02 | 4 | 0.01 | 1 | 0.01 |
| Carbohydrate metabolic process | 29 | 0.04 | 14 | 0.02 | 1 | 0 | 0 | 0 |
| Cell cell signaling | 16 | 0.02 | 13 | 0.02 | 2 | 0.01 | 0 | 0 |
| Cell cycle | 40 | 0.06 | 55 | 0.07 | 23 | 0.07 | 9 | 0.05 |
| Cell differentiation | 72 | 0.1 | 63 | 0.08 | 17 | 0.05 | 10 | 0.06 |
| Cell projection organization | 30 | 0.04 | 28 | 0.04 | 8 | 0.03 | 4 | 0.02 |
| Cellular component morphogenesis | 28 | 0.04 | 28 | 0.04 | 6 | 0.02 | 3 | 0.02 |
| Cellular component movement | 32 | 0.05 | 19 | 0.02 | 4 | 0.01 | 3 | 0.02 |
| Extracellular matrix organization | 6 | 0.01 | 2 | 0 | 0 | 0 | 0 | 0 |
| Homeostatic process | 39 | 0.06 | 30 | 0.04 | 12 | 0.04 | 4 | 0.02 |
| Lipid metabolic process | 30 | 0.04 | 21 | 0.03 | 3 | 0.01 | 3 | 0.02 |
| Protein catabolic process | 22 | 0.03 | 38 | 0.05 | 15 | 0.05 | 8 | 0.04 |
| Protein folding | 29 | 0.04 | 15 | 0.02 | 3 | 0.01 | 2 | 0.01 |
| Regulation apoptosis | 56 | 0.08 | 56 | 0.07 | 22 | 0.07 | 10 | 0.06 |
| Ribonucleoprotein complex assembly | 13 | 0.02 | 17 | 0.02 | 8 | 0.03 | 5 | 0.03 |
| Ribosome biogenesis | 15 | 0.02 | 28 | 0.04 | 22 | 0.07 | 15 | 0.08 |
| Translation | 54 | 0.08 | 103 | 0.13 | 78 | 0.24 | 60 | 0.34 |
| Transport | 97 | 0.14 | 116 | 0.15 | 41 | 0.13 | 17 | 0.1 |

# = total number of scores, freq = frequency of genes participating in a specific biological process
